# COVID-19 Contact Tracing Highlights Disparities: Household Size and Low-English Proficiency

**DOI:** 10.1089/heq.2021.0148

**Published:** 2022-04-27

**Authors:** Marisa E. Giglio, Matthew Pelton, Ae Lim Yang, Akshilkumar Patel, Lindsay K. Buzzelli, Jacob Schick, Anna Ssentongo, Zizhong Tian, Casey A. Ryan, Nina Razavi, Benjamin Fredrick

**Affiliations:** ^1^ Pennsylvania State College of Medicine, Hershey, Pennsylvania, USA.; ^2^ Department of Public Health, Penn State Hershey College of Medicine, Hershey, Pennsylvania, USA.; ^3^ Department of Family Medicine, Penn State Health Milton S. Hershey Medical Center, Hershey, Pennsylvania, USA.

**Keywords:** COVID-19, health care disparities, preventative medicine, intervention

## Abstract

Although it is known that coronavirus disease 2019 (COVID-19) disproportionately affects racial and ethnic minorities, our study characterizes the connection between COVID-19 susceptibility and both limited English proficiency (LEP) and large household size. We examined demographic and social data for 1130 individuals who tested positive for or were exposed to COVID-19. Analysis revealed that LEP persons were 3.2 times as likely to report difficulty obtaining supplies for quarantine. Individuals in large households were 1.9 times as likely to report difficulty obtaining supplies for quarantine and 2.0 times as likely to report inability to quarantine. This study, therefore, informs interventions targeted to these populations.

## Introduction

The novel coronavirus disease 2019 (COVID-19) has led to a global pandemic that disproportionately affects certain populations traditionally disenfranchised by the health care system.^1,2^ Although some social determinants of health including social, environmental, and economic contributing factors have been described in the literature, we suspect an important overlap between COVID-19 susceptibility and patients with limited English proficiency (LEP) and large household sizes.^1–3^

In the United States, ∼25 million individuals identify as a member of the LEP population.^4,5^ Patients with LEP experience poor patient–clinician communication and worse health outcomes compared with their native English-speaking counterparts.^4,6^ One recent study found that non-English primary languages were the strongest predictor of risk of COVID-19 infection among age, race, ethnicity, language, income, and living conditions.^1^ In addition, poor housing conditions, including overcrowding and high density, result in increased transmission of COVID-19.^7^ Early data approximated the rate of COVID-19 infection within a household to range from 16.3%^8^ to 38%^9^ that is greater than that of severe acute respiratory syndrome, middle east respiratory syndrome, and influenza A (2009).

Patients with LEP have faced numerous health care disparities throughout the COVID-19 pandemic, however, there is limited data that investigate the relationship between LEP and large household size and how communities that fall into both of these categories have been disproportionately affected by the pandemic. We sought to further examine the barriers to health care access, quarantine, and isolation that are faced by large households and the LEP population during the pandemic.

## Methods

A retrospective review was performed using data collected by the Hershey Medical Center Contact Tracing Team from March to August 2020. Information regarding 1130 COVID-19–positive patients and their contacts was obtained. Data including individual demographics, interventions received, work excuses given, living with a high-risk individual, household size, utilization of the health care system, primary care utilization, insurance status, and self-reported ability to quarantine, including physical space in the home and ability to remain in the home for the quarantine period, were collected for each patient and their contacts.^10^

We divided all individuals into two groups based on preferred language, English proficient (EP), or LEP, defined as persons who are not fluent in the English language. In addition, individuals were placed into groups based on household size. Individuals living in households with less than three people were labeled as living in a small household and those living with three or more people were labeled as living in a large household. Chi-squared analyses and unadjusted odds ratios (ORs) were calculated to examine differences in EP versus LEP persons and persons in small households versus persons in large households.

In addition, multivariable logistic regression analyses were performed to evaluate the effects of nine potential predictors on two outcomes of interest. The outcome variables of interest are difficulty obtaining supplies, and interventions received, both of which are binary variables. The candidate-independent variables include one continuous variable (age) and eight categorical variables (household size, gender, ethnicity, language, primary care physician status, insurance status, utilization of health care system, and pre-existing conditions). The most statistically pertinent predictors for each outcome were screened, multiple logistic regression models were built, and interactive effects were tested.

For each outcome, a univariable logistic regression using nine candidate predictors to exclude nonsignificant predictors was run. Independent variables with *p*-value <0.1 were added into the multiple logistic regression model. After the multivariable models were constructed, any possible pairwise interaction terms were attempted to add into the model one at a time, and the interaction effects would be tested by likelihood ratio tests. For each outcome model, the estimated OR and 95% confidence intervals (CIs) were calculated. This project was reviewed by the institution's Human Subject Protection Office and determined to be consistent with quality improvement and not research.

## Results

### EP versus LEP

Of the 1130 cases and contacts, 57.0% (*n*=644) were EP, 20.4% (*n*=231) were LEP, and 22.6% (*n*=255) were not reported. The mean household size for EP persons was 2.08 individuals (95% CI: 1.9–2.3) and significantly smaller than the mean household size for LEP persons, which was 3.10 (95% CI: 2.7–3.4); therefore, EP individuals were 2.2 times as likely to live in a small household (95% CI: 1.2–3.1). LEP persons were 3.2 times as likely to report having difficulty obtaining supplies necessary for quarantine (95% CI: 2.0–5.1), and 5.9 times as likely to receive interventions from our contact tracing team (95% CI: 3.6–9.7) when compared with EP persons.

LEP persons were more likely to identify as Asian or multiple races (*p*=0.001), identify as Hispanic (*p*<0.0001), and report having public insurance or being uninsured (*p*=0.001). A full comparison of EP and LEP individuals is presented in Table 1. There was no statistically significant difference in gender (*p*=0.21), doctor's notes given for quarantine (*p*=0.81), living with a high-risk individual (*p*=0.87), or ability to quarantine at home (*p*=0.97) between the two groups.

**Table 1.** Demographic and Health Care Accessibility Differences Between English Proficient Versus Limited English Proficiency

Characteristics	EP vs. LEP		*p*
	EP, %	LEP, %	
Sample, % (*n*)	57 (644)	20.4 (231)	
Age (mean)	39.9	38.6	
Race			0.001
Caucasian	64.6	11.2	
African American	13.0	2.6	
Asian	6.6	34.9	
Other (multiple races)	15.7	51.3	
Ethnicity			<0.0001
Non-Hispanic	86.3	61.8	
Hispanic	13.7	38.2	
Gender			0.21
Male	42.5	47.6	
Female	57.5	52.4	
Ability obtaining supplies necessary for quarantine			<0.0001
No difficulty obtaining supplies	89.4	72.4	
Difficulty obtaining supplies	10.7	27.6	
Received interventions			<0.0001
Yes	9.2	39.4	
No	90.8	60.6	
Insurance status			0.001
Private	69.2	44.6	
Public	18.3	29.5	
Uninsured	9.1	21.1	
Not reported	3.3	4.8	

EP, English proficient; LEP, limited English proficiency.

### Small versus large household size

Data on household size were available for 798 (70.6%) of individuals. Of those included, 57.3% (*n*=457) were small households and 42.7% (*n*=341) were large households. Individuals in large households were 1.9 times more likely to report difficulty obtaining supplies necessary for quarantine (95% CI: 1.2–3.0), 2.3 times as likely to report living with a high-risk individual as compared with small households (95% CI: 1.7–3.2), 1.9 times as likely to report being unable to quarantine at home (95% CI: 1.2–3.4), and 1.7 times as likely to receive interventions from our contact tracing team (95% CI: 1.0–2.7) as compared with small households.

In addition, individuals in large households were more likely to report being of Asian descent as compared with small household individuals (*p*<0.001). A full comparison of household sizes is presented in Table 2. There was no statistically significant difference in gender (*p*=0.37), ethnicity (*p*=0.17), doctors notes given for quarantine (*p*=0.92), insurance status (*p*=0.28), or utilization of the health care system within the past 12 months (*p*=0.15) between the two groups.

**Table 2.** Demographic and Health Care Accessibility Differences Between Large Versus Small Household Individuals

Characteristics	Small households vs. large households		*p*
	Small households, %	Large households, %	
Sample, % (*n*)	57.3 (457)	42.7 (341)	
Age (mean)	40.1	39.3	
Race			<0.001
Caucasian	61.4	40.4	
African American	10.6	7.0	
Asian	4.6	24.9	
Other	23.4	27.7	
Ethnicity			0.17
Non-Hispanic	62.0	54.7	
Hispanic	38.0	45.3	
Gender			0.37
Male	41.8	45.0	
Female	58.2	55.0	
Ability obtaining supplies necessary for quarantine			0.003
No difficulty obtaining supplies	90.1	82.6	
Difficulty obtaining supplies	9.9	17.4	
Living with a high-risk individual			<0.0001
No	69.7	49.8	
Yes	30.3	50.2	
Ability to quarantine at home			0.01
Able to quarantine at home	93.5	87.9	
Unable to quarantine at home	6.5	12.1	
Received interventions			0.03
Yes	10.9	16.9	
No	89.1	83.1	

### Multivariate regression

The multiple logistic regression model for difficulty obtaining supplies showed large household size (OR: 1.9, 95% CI: 1.2–3.0) and LEP (OR: 3.2, 95% CI: 2.0–5.1) are significant positive predictors. The interactive effect between household size and English proficiency was not statistically significant (*p*=0.47). For interventions received, LEP (OR: 5.9, 95% CI: 3.6–9.7), large household (OR: 1.7, 95% CI: 1.0–2.7), and one unit increase in age (OR: 3.9, 95% CI: 1.1–19.9) are positive predictors for interventions received. The interactive effect between household size and language was not statistically significant (*p*=0.13). For all multivariable models, no interaction effect was detected between any selected predictors. The full multivariable logistic regression is presented in Table 3.

**Table 3.** Results for Multivariable Logistic Regression Analyses on Outcomes of Interest

	Coefficient estimate	OR	95% CI	*p*
Whether having difficulty to obtain supplies or not				
Household size Large vs. small	0.56	1.9	1.2–3.0	0.003
Language LEP vs. EP	0.99	3.2	2.01–5.13	<0.001
Whether receiving interventions or not				
Household size Large vs. small	0.70	1.7	1.0–2.7	0.04
Language LEP vs. EP	0.39	5.9	3.6–9.7	<0.0001
Age in years 1-year increase	2.17	3.9	1.1–19.9	0.003

CI, confidence interval; OR, odds ratio.

## Discussion

Patients with LEP have faced numerous health care disparities throughout the COVID-19 pandemic. Our study provides further evidence that patients with LEP and patients in large households are at risk of noncompliance with quarantine recommendations. Studies have already shown that LEP patients are more likely to be admitted for COVID-19 infection, which may denote increased disease severity in this population.^11^ We found that persons with LEP experienced greater difficulty obtaining supplies necessary for quarantine and this may partially explain the increased risk of COVID-19 infection in this population.

If these individuals break quarantine more frequently, they are put at higher risk of contracting COVID-19 and place their contacts at higher risk of infection in turn. Furthermore, LEP persons in our study lived with more individuals on average than EP persons, increasing their potential for COVID-19 exposure. Our finding that LEP persons required more support from our team may be a product of the increased number of persons per household or due to difficulty accessing the available resources.

Patients from larger households tested for COVID-19 are more likely to be COVID-19 positive, to subsequently be admitted to a hospital, and, ultimately, to die from the disease.^12,13^ Our analysis showed increased risk for persons in large households and with LEP. Our finding that persons in large households had more difficulty obtaining supplies, more frequently lived with high-risk individuals, and were less able to quarantine at home that could explain the increased infection rate and severity of COVID-19 in this population. In addition, our finding that individuals in large households required more support from our team may be a product of these risk factors.

The primary strength of this study is the thorough needs assessment conducted by our contact tracing team. Since the study required self-reporting, a main limitation of this study is potential for response bias. In addition, a large proportion of the sample was excluded from the analysis due to lack of response to the language preference question. This study was conducted from a central Pennsylvania population and, therefore, may not be representative of the entire United States.

Our findings contribute to the existing evidence about the health disparities experienced by persons living in large households and those with LEP in the COVID-19 pandemic. Increased barriers that these populations face, such as difficulty obtaining supplies, may contribute to higher positivity rates by increasing their potential exposures. These populations may benefit from targeted interventions to provide education in preferred languages, quarantine strategies within larger households, and appropriate supplies to support quarantine.

## Abbreviations Used

CI: confidence interval
COVID-19: coronavirus disease 2019
EP: English proficient
LEP: limited English proficiency
OR: odds ratio
